# Phase 1 dose escalation trial of the selective adenosine A2B antagonist PBF-1129 in patients with metastatic non-small cell lung cancer

**DOI:** 10.1007/s10637-025-01591-y

**Published:** 2025-12-03

**Authors:** Dwight H. Owen, Ruohan Wu, Lai Wei, Mikhail Dikov, Shankar Suman, Po-Lan Su, Joseph Amann, Shuxiao Guan, Daniel Spakowicz, Catherine Schweitzer, Carly Pilcher, Michael B. Smith, Sarah Ferguson, Julio Castro Palomino, Santiago Figueroa Pérez, Erin M. Bertino, Peter G. Shields, Kai He, Carolyn J. Presley, Gregory A. Otterson, William E. Carson, David P. Carbone

**Affiliations:** 1 Division of Medical Oncology, Department of Internal Medicine, The Ohio State University Comprehensive Cancer Center, 1800 Cannon Drive, 12th Floor, Columbus, OH USA; 2 Pelotonia Institute for Immuno-Oncology, The Ohio State University Comprehensive Cancer Center, Columbus, OH USA; 3 Center for Biostatistics, The Ohio State University Wexner Medical Center, Columbus, OH USA; 4 Department of Internal Medicine, National Cheng Kung University Hospital, College of Medicine, National Check Kung University, Tainan, Taiwan; 5 Clinical Trials Office, The Ohio State University Comprehensive Cancer Center, Columbus, OH USA; 6 Department of Pharmacy, The Ohio State University Comprehensive Cancer Center, Columbus, OH USA; 7 Palobiofarma S.L., Pamplona, Spain; 8 Department of Oncology, Mayo Clinic, Rochester, MN USA

**Keywords:** Non-small cell lung cancer, Phase 1 clinical trial, Adenosine receptor, Immunotherapy

## Abstract

The adenosinergic pathway represents a critical immunometabolic checkpoint within the tumor microenvironment of non-small cell lung cancer (NSCLC), contributing to immune suppression and therapeutic resistance. PBF-1129, an oral, selective A2B adenosine receptor (A2BAR) antagonist, was evaluated in a phase 1, open-label, dose-escalation trial (NCT03274479) in patients with advanced/metastatic NSCLC who had progressed on standard therapies. All patients had previously received chemotherapy and immune checkpoint blockade. Twenty-one patients received escalating doses (40–320 mg once daily), with no dose-limiting toxicities observed. The most frequently reported treatment related adverse events of any grade were lymphocytopenia (*n* = 8, 38.1%), vomiting (*n* = 8, 38.1%), anorexia (*n* = 6, 28.5%), and fatigue (*n* = 6, 28.5%). PBF-1129 showed dose-proportional pharmacokinetics and maintained plasma concentrations above the 90% maximal inhibitory concentration (IC_90_) of A2BAR at 320 mg for 24 h, which was determined to be the recommended phase 2 dose (RP2D). Best response was stable disease in 3 out of 21 patients including 2 of 6 treated at RP2D. Immunophenotyping revealed post-treatment reductions in programmed cell death protein 1 (PD-1) expression on CD8⁺ T cells, which correlated with improved survival. The reduction of PD-1 expression on CD4⁺ T cells and decreased myeloid-derived suppressor cells were also associated with better outcomes. These findings suggest PBF-1129 is safe and modulates the systemic immune parameters, warranting further evaluation in combination with immune checkpoint blockade.

**Supplementary information:**

The online version contains supplementary material available at 10.1007/s10637-025-01591-y.

## Introduction

Despite recent progress in treating metastatic non-small cell lung cancer (mNSCLC), only a subset of tumors respond to currently available immunotherapy treatments; those that do respond eventually develop treatment resistance. A possible barrier to durable responses is the immunosuppressive tumor microenvironment (TME), which involves several immunosuppressive factors and cells. To develop an effective immunologic approach to treat lung cancer, strategies to induce immune activation and inhibit suppressive mechanisms may be required [1]. The TME is characterized by hypoxia, acidic extracellular pH, altered redox potential, low nutrient levels, high increased interstitial fluid pressure, and elevated adenosine triphosphate (ATP) levels. Extracellular ATP acts as an alarm signal, which promotes immunogenic cell activity and facilitates antitumor immune responses [2]. The ATP in the TME is actively converted to the immunosuppressive metabolite adenosine by ATP-hydrolyzing ectoenzymes including CD39 and CD73, which are highly expressed in tumors and subsets of immune cells [1, 3–7]. Targeting this pathway represents an emerging strategy to overcome immune resistance in NSCLC [8].

Extracellular adenosine is an established tumor protective and immunosuppressive factor with elevated levels in the interstitium of solid tumors [9–12]. It has dual functions as a metabolite and a signaling molecule. Extracellular adenosine levels in normal tissue are at low picomolar concentrations but become significantly elevated in response to ischemic or inflammatory damage [3, 12]. Adenosinergic signaling has emerged as a powerful immunometabolic checkpoint in tumors, where its elevated levels play a tumor-protective and immunosuppressive role [9–11, 13]. For example, the CD73-targeting monoclonal antibody oleclumab has demonstrated activity in multiple studies in NSCLC [8]. Adenosine activates pathways that promote tissue adaptation in hypoxic environments including enhancing metabolic ischemia tolerance and dampening inflammation. High expression of adenosine-generating enzymes is strongly associated with poor clinical outcomes and resistance to immunotherapy for patients with different types of cancer [14–17].

In addition to monoclonal antibodies that target extracellular ectoenzymes, preclinical studies on the use of small-molecule inhibitors to interfere with adenosine generation or signaling through A2A and A2B adenosine receptors (A2BAR) have been shown to relieve immunosuppression, reducing stress in the TME by decreasing expression of key adenosine-generating enzymes. A2BAR blockade enhances antitumor immunity through a reduction in myeloid-derived suppressor cell (MDSC) differentiation and an enhancement of the capacity of dendritic cells (DCs) to evoke antitumor T-cell responses. We previously demonstrated that adenosine binding to A2BAR interferes with the differentiation of monocytes to DCs and alters their phenotype toward cells secreting pro-angiogenic and immunosuppressive factors [18–21]. Triggering of A2A and A2B receptors diminishes the capacity of DCs to prime Th1 immune responses [22, 23]. We also found that A2B receptor blockade enhanced antitumor immunity partly through an enhancement of the capacity of DCs to evoke antitumor T-cell responses [18, 19, 23]; in addition, we saw enhanced antitumor activity in vivo when A2BAR blockade with PBF-1129 was combined with anti-programmed cell death protein 1 (PD-1) therapy [24]. PBF-1129 is an oral potent and selective A2BAR antagonist. Here, we present results from a dose-escalation phase 1 trial of PBF-1129 in patients with NSCLC (ClinicalTrials.gov: NCT03274479).

## Methods

NCT03274479 is an open-label, single-site, dose-escalation phase 1 trial of PBF-1129 monotherapy in patients with NSCLC whose cancer has progressed despite treatment with standard-of-care therapies. The study was approved by The Ohio State University Institutional Review Board (#2018C0019) and was done in accordance with local and/or national regulations and the ethical principles in the Declaration of Helsinki. All patients provided written informed consent before entering the study.

### Patients

Eligible patients were ≥ 18 years of age and had histologically or cytologically confirmed advanced or metastatic NSCLC (squamous or nonsquamous histology) with progression on other treatments known to confer benefit. There was no limit to lines of prior therapy. Other inclusion criteria included measurable disease as determined by Response Evaluation Criteria in Solid Tumors (RECIST) version 1.1, an Eastern Cooperative Oncology Group (ECOG) performance status of ≤ 2, and adequate organ function. Patients were ineligible if they had received other systemic anticancer chemotherapy within 21 days of study or had unresolved grade 2 or higher toxicity from previous treatment. Asymptomatic central nervous system metastases were permitted; however, patients with symptomatic lesions, carcinomatous meningitis, active infection requiring therapy, history of autoimmune disease, diagnosis of immunodeficiency or receipt of immunosuppressive therapy ≤ 7 days before treatment allocation, or use of immunosuppression medication were excluded.

### Study design

This was a phase 1, open-label, single-institution, multiple-dose, dose-escalation study. Patients received escalating doses of PBF-1129 orally daily (40 mg, 80 mg, 160 mg, 320 mg) in 28-day cycles until disease progression per RECIST v1.1 or intolerability. Starting dose levels were chosen based on single dose and multiple dose phase 0 studies in healthy volunteers. The dose escalation was conducted using the 3 + 3 method. PBF-1129 was administered fasting (2 h before and/or after meals), once daily continuously. The primary objectives were (1) safety and tolerability as defined by the occurrence of dose-limiting toxicities (DLTs) and (2) determination of the maximum tolerated dose (MTD). Pharmacokinetics (PK), objective response rate (ORR), progression-free survival (PFS), and overall survival (OS) were secondary endpoints.

### Assessments

Patients were monitored for DLTs during the first 28-day treatment cycle. DLT evaluable patients include all subjects who complete the safety follow-up through the DLT evaluation period or who experience a DLT during the DLT evaluation period. A DLT was defined as any grade ≥ 3 nonhematologic or grade 4 hematologic adverse event (AE) likely related to the study drug and occurring during the DLT period. The following exceptions were not considered as a DLT: a grade 3 decrease in lymphocyte count or increase in gamma glutamyl transferase (GGT) that downgraded to grade ≤ 2 within 7 days after onset of the event and resolved to grade ≤ 1 or baseline within 14 days; any grade 3 endocrinopathy that was managed with or without systemic corticosteroid therapy and/or hormone replacement therapy in an asymptomatic patient; or a grade 3 inflammatory reaction attributed to a local antitumor response (for example, an inflammatory reaction at sites of metastatic disease, lymph nodes, etc.). DLT grading followed the guidelines provided in the Common Terminology Criteria for Adverse Events (CTCAE) version 4.03.

### Pharmacokinetics

Plasma samples for analyzing the PK of PBF-1129 were collected on day 1 of cycles 1 and 2 (predose, and at 10 min, 60 min, 2 h, and 4 h post-medication).

### Immunophenotyping and cytokine analysis

Peripheral blood samples were collected for immunophenotyping and cytokine analysis at the following time points: day 1 of cycle 1 (C1D1), day 1 of cycle 2 (C2D1), and end of treatment (EOT). Plasma samples were collected by centrifugate, and peripheral blood mononuclear cells (PBMCs) were isolated by Ficoll-Paque (Fisher Scientific) gradient followed by cryopreservation. For immunophenotyping purposes, 21 cell surface markers and 4 intracellular markers were evaluated by flow cytometry (3-laser Northern Lights, Cytek). Thirteen tumor-associated cytokines were detected by the LEGENDplex cytokine detection kit (BioLegend) following the manufacturer’s protocol.

### Statistics

The safety and efficacy analysis set included all subjects who received at least 1 dose of PBF-1129. The MTD was defined as the highest dose level (DL) at which 6 patients were treated and at most 1 patient had a DLT in the DLT assessment period (28 days). Based on a review of the non-DLT AE data, laboratory parameters, and other study data (for example, PK, pharmacodynamics [PD], and efficacy), the recommended phase 2 dose (RP2D) was determined (a DL less than or equal to the MTD). Toxicities were summarized by grade using frequency and percentage.

For each cohort or DL, summaries of all important descriptors (for example, age, gender, and response rate) were produced using descriptive statistics such as mean and standard deviation for measured continuous variables and frequencies and percentages for categorical variables. We also used histograms and box-plots to understand aspects of data quality and overall characteristics of the data. ORR was defined as confirmed complete response (CR) or partial response (PR) based on modified RECIST v1.1. Response rates were calculated with an exact binomial 95% confidence interval (CI). PFS was measured from the start of treatment until the documentation of disease progression or death due to any cause, whichever occurred first. OS was determined as the time from the start of treatment with PBF-1129 until death due to any cause. Median survivals were estimated using the Kaplan–Meier method with 95% CI. The survival curves were compared using the log-rank test.

For immunophenotyping and cytokine data, paired t-tests or the Wilcoxon rank sum test were used to compare pre- vs. post-treatment samples. Results of immunophenotyping were compared for each individual patient pre-treatment (C1D1) vs. post-treatment (C2D1 or EOT). Cytokine levels were compared between C1D1, C2D1, and EOT across the 4 dosage levels. The OS was compared between patients with greater versus lesser reductions in cytokine levels or PBMC composition, based on the median value of reduction. These analyses were conducted in SAS version 9.4 (SAS Institute, Cary, NC) and R (version 4.2.0, R Foundation for Statistical Computing, Vienna, Austria). Statistical significance was concluded at a *P* value of < 0.05.

## Results

### Patient characteristics

A total of 21 patients were enrolled in the study. Baseline characteristics are shown in Table 1, including median age (61 years, interquartile range [IQR], 49–75); sex (9 patients [42.9%] were female); and disease histology (12 patients [57.1%] with adenocarcinoma, 7 [33.3%] with squamous cell carcinoma, and 1 each [4.8%] with adenosquamous carcinoma and NSCLC not otherwise specified [NOS]). The median number of prior lines of therapy was 4 (IQR, 3–6). All patients had previously received immune checkpoint inhibitors (ICIs) targeting PD-1 or programmed cell death ligand 1 (PD-L1), as well as chemotherapy; 4 patients (19.0%) had also received prior anti-cytotoxic T-lymphocyte-associated protein 4 (CTLA-4) therapy. PD-L1 expression was negative in 6 patients (28.6%), positive in 12 (57.1%), and unknown in 3 (14.3%). *TP53* mutations were the most prevalent mutation detected in 9 patients (42.9%), followed by *KRAS* (8/21, 38.1%), *CDKN2A* (2/21, 9.5%), *STK11* (2/21, 9.5%), and *FGFR* (2/21, 9.5%) (Supplementary Fig. 1).

**Table 1:** Baseline Characteristics

Characteristics	No. of patients (%) *N* = 21
Age	
Median (IQR), years	61 (55–65)
Sex	
Male Female	12 (57.1%) 9 (42.9%)
Histology	
Adenocarcinoma Squamous cell carcinoma Adenosquamous carcinoma NSCLC NOS	12 (57.1%) 7 (33.3%) 1 (4.8%) 1 (4.8%)
PD-L1 expression	
≥ 50%	5 (23.8%)
1–49% < 1% N/A/unknown	7 (33.3%) 6 (28.6%) 3 (14.3%)
Median line of therapy (IQR) Prior anti-PD-1/L1 Prior anti-CTLA-4	4 (3–6) 21 (100%) 4 (19%)

*IQR* interquartile range, *NOS* not otherwise specified

### Safety and tolerability

During the study period, no DLTs were observed at any dose level; consequently, the MTD was not reached. Four patients were enrolled at dose level 1 (40 mg QD), 4 patients at dose level 2 (80 mg QD), 7 patients at dose level 3 (160 mg QD), and 6 patients at dose level 4 (320 mg QD) as 1, 1, and 4 patients, respectively, were not DLT-evaluable and were replaced. Treatment related adverse events (TRAE) have been summarized for all 21 patients who received at least one dose of PBF-1129 in Table 2. Grade ≥ 3 TRAEs occurred in 3 patients (14.3%), including lymphocytopenia (*n* = 2, 9.6%), hyponatremia (*n* = 1, 4.9%), hypertension (*n* = 1, 4.9%), and encephalopathy (*n* = 1, 4.9%). The most frequently reported TRAEs of any grade were lymphocytopenia (*n* = 8, 38.1%), vomiting (*n* = 8, 38.1%), anorexia (*n* = 6, 28.5%), and fatigue (*n* = 6, 28.5%).

**Table 2:** Treatment-related Adverse Events

Adverse Event	Toxicity Category	N	%	N	%	N	%
		G1&2	G1&2	G3 +	G3 +	total	total
Vomiting	Gastrointestinal disorders	8	38%	0	0%	8	38%
Fatigue	General disorders and administration site conditions	7	33%	1	5%	8	38%
Lymphocyte count decreased	Investigations	6	29%	2	10%	8	38%
Nausea	Gastrointestinal disorders	6	29%	0	0%	6	29%
Anorexia	Metabolism and nutrition disorders	6	29%	0	0%	6	29%
Diarrhea	Gastrointestinal disorders	4	19%	0	0%	4	19%
Aspartate aminotransferase increased	Investigations	4	19%	0	0%	4	19%
Anemia	Blood and lymphatic system disorders	3	14%	0	0%	3	14%
Alkaline phosphatase increased	Investigations	3	14%	0	0%	3	14%
Weight loss	Investigations	3	14%	0	0%	3	14%
Myalgia	Musculoskeletal and connective tissue disorders	3	14%	0	0%	3	14%
Dizziness	Nervous system disorders	3	14%	0	0%	3	14%
Constipation	Gastrointestinal disorders	2	10%	0	0%	2	10%
Malaise	General disorders and administration site conditions	2	10%	0	0%	2	10%
Arthralgia	Musculoskeletal and connective tissue disorders	2	10%	0	0%	2	10%
Blurred vision	Eye disorders	1	5%	0	0%	1	5%
Gastroesophageal reflux disease	Gastrointestinal disorders	1	5%	0	0%	1	5%
Alanine aminotransferase increased	Investigations	1	5%	0	0%	1	5%
Electrocardiogram QT corrected interval prolonged	Investigations	1	5%	0	0%	1	5%
Lipase increased	Investigations	1	5%	0	0%	1	5%
Serum amylase increased	Investigations	1	5%	0	0%	1	5%
White blood cell decreased	Investigations	1	5%	0	0%	1	5%
Hyperuricemia	Metabolism and nutrition disorders	1	5%	0	0%	1	5%
Hypoalbuminemia	Metabolism and nutrition disorders	1	5%	0	0%	1	5%
Hypokalemia	Metabolism and nutrition disorders	1	5%	0	0%	1	5%
Hypomagnesemia	Metabolism and nutrition disorders	1	5%	0	0%	1	5%
Hyponatremia	Metabolism and nutrition disorders	0	0%	1	5%	1	5%
Flank pain	Musculoskeletal and connective tissue disorders	1	5%	0	0%	1	5%
Concentration impairment	Nervous system disorders	1	5%	0	0%	1	5%
Encephalopathy	Nervous system disorders	0	0%	1	5%	1	5%
Headache	Nervous system disorders	1	5%	0	0%	1	5%
Hypersomnia	Nervous system disorders	1	5%	0	0%	1	5%
Peripheral sensory neuropathy	Nervous system disorders	1	5%	0	0%	1	5%
Insomnia	Psychiatric disorders	1	5%	0	0%	1	5%
Cough	Respiratory, thoracic and mediastinal disorders	1	5%	0	0%	1	5%
Rash maculo-papular	Skin and subcutaneous tissue disorders	1	5%	0	0%	1	5%
Hypertension	Vascular disorders	0	0%	1	5%	1	5%

### Pharmacokinetics

**Fig. 1:**
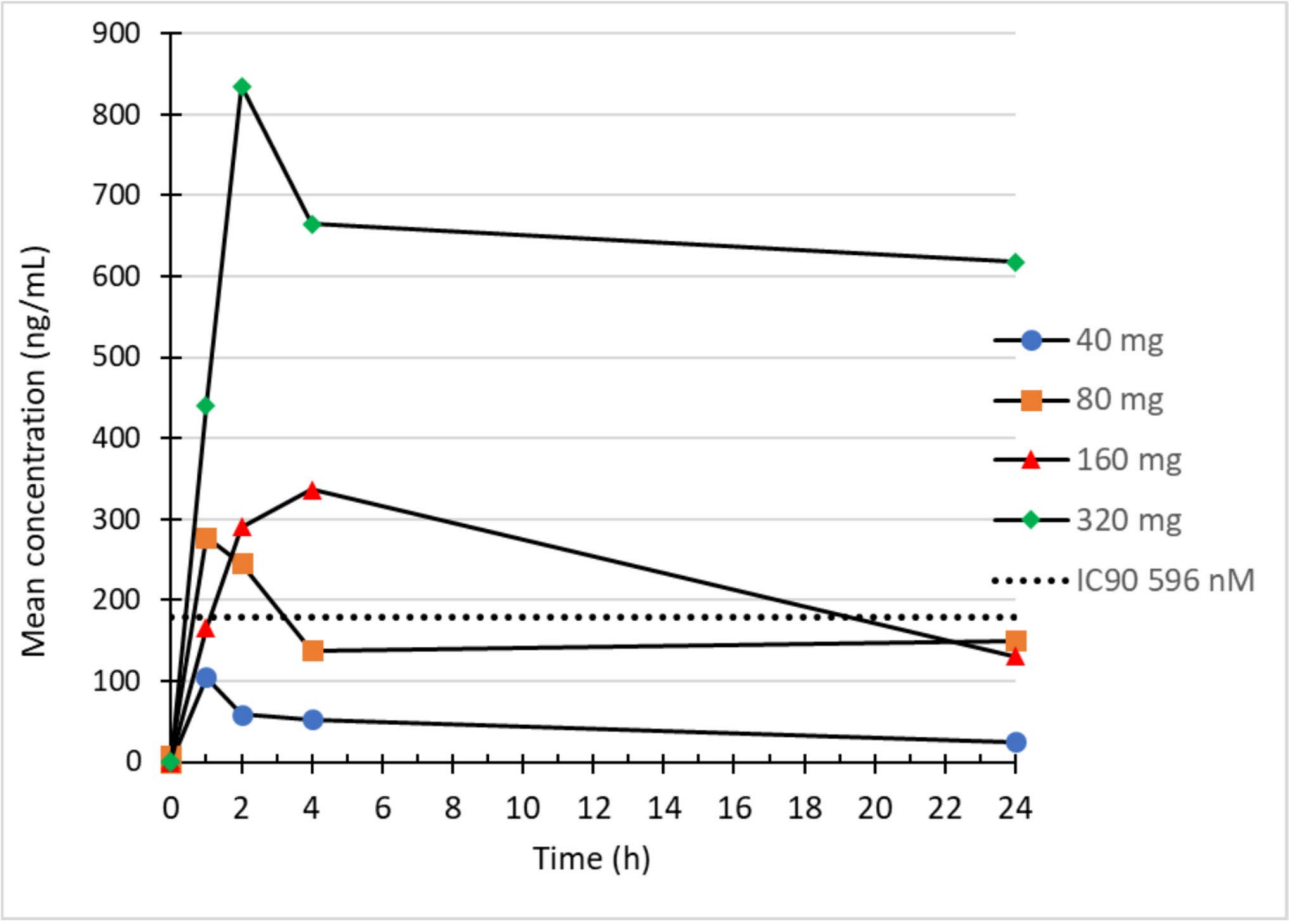
Mean serum concentrations of PBF-1129 following once-daily oral administration in patients with mNSCLC. PK analysis showed a dose-dependent increase in drug exposure, with the median maximum plasma concentration rising from 150 ng/mL at 40 mg to 800 ng/mL at 320 mg. The drug had a moderate half-life of over 10 hours. At 320 mg, plasma levels remained above the IC_90_ (dotted line) for the A2BAR antagonist for 24 hours. PBF-1129 binds to the adenosine A2b receptor with an IC90 of 596 nM (179 ng/mL)

PK analysis demonstrated a dose-dependent increase in drug exposure, with the median maximum plasma concentration (C_max_) ranging from 150 ng/mL at the 40-mg dose (DL 1) to 800 ng/mL at the 320-mg dose (DL 4). The drug exhibited a moderate half-life exceeding 10 h (Fig. 1). Notably, the 320-mg dose (DL 4) maintained free plasma concentrations of PBF-1129 above the 90% maximal inhibitory concentration (IC_90_) for the A2BAR over a 24-h period.

### Efficacy

**Fig. 2:**
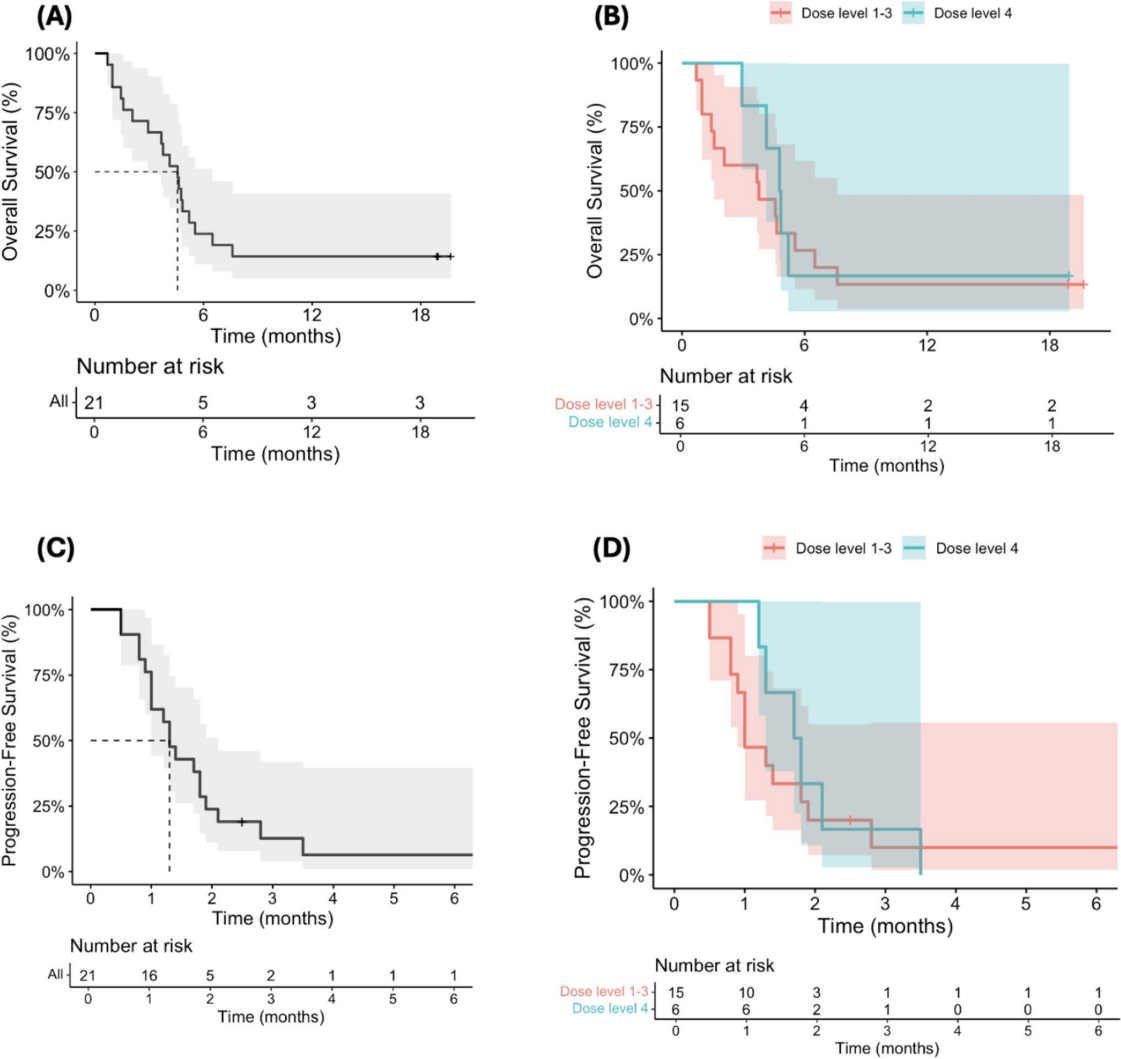
Kaplan-Meier survival analysis depicting OS and PFS of mNSCLC patients treated with PBF-1129.** A**. The median OS was 4.6 months (95% confidence interval [CI], 2.1–5.2.1.2) **B**. with no significant difference observed between patients receiving dose levels 1–3 and those receiving dose level 4. **C**. The median PFS was 1.5 months (95% CI, 1.0–1.9.0.9), **D**. with similar outcomes noted between the lower (DLs 1–3) and higher (DL 4) dose groups

The median OS was 4.6 months (95% CI, 2.1–5.2) (Fig. 2A), with no significant difference observed between patients receiving DLs 1–3 (40, 80, and 160 mg) and those receiving DL 4 (320 mg) (Fig. 2B). The median PFS was 1.5 months (95% CI, 1.0–1.9) (Fig. 2C), with similar outcomes noted between the lower (DLs 1–3) and higher (DL 4) dose groups (Fig. 2D). The best overall response was stable disease (SD) in 3 patients (14%) while no patients achieved a PR or CR.

Patients with adenocarcinoma histology had a median OS of 4.80 months (95% CI, 4.57-not reached [NR]), which was longer than that of patients with non-adenocarcinoma histology (3.67 months; 95% CI, 0.97-NR) (*P* = 0.019) (Supplementary Figure S1D). However, no significant differences in OS were observed based on age (≥ 60 vs. < 60 years) (Supplementary Figure S1A), sex (male vs. female) (Supplementary Figure S1B), or PD-L1 expression levels (≥ 50% vs. < 50%) (Supplementary Figure S1C).

### Cytokine analysis

Serum cytokine levels from 39 samples were evaluated from 16 patients. Significant increases in IL-4, IL-2, TNF-α, IL-17A, and IL-6 were observed at EOT compared to C2D1 or C1D1, after adjusting for dosage level and time point using a linear mixed-effects model. Similarly, IL-4 levels at EOT were significantly higher than those at C1D1. These findings are consistent with previously generated data from animal models of A2BAR inhibition. Patients with higher baseline IL-4 (> 2.12 pg/ml) or TNF-α (> 0.76 pg/ml) levels exhibited a median OS of 4.57 months (95% CI, 2.07-NR), which was significantly shorter than that of patients with lower baseline IL-4 or TNF-α levels (6.40 months; 95% CI, 4.83-NR; *P* = 0.025) (Supplementary Figures S2A and S2B). In contrast, baseline expression levels of IP-10, MCP-1, IL-17A, IL-6, and IL-2 were not associated with survival (Supplementary Figures S2C-S2G).

### Peripheral immunophenotyping analysis

Among the 21 trial participants, baseline samples were available for 18 patients, with paired pre-treatment (baseline) and post-treatment (EOT) samples collected from 9 patients. Peripheral blood samples were obtained at 3 time points: C1D1, C2D1, and EOT. A total of 30 PBMC samples were analyzed using flow cytometry (panel details provided in Supplementary Table S1).

**Fig. 3:**
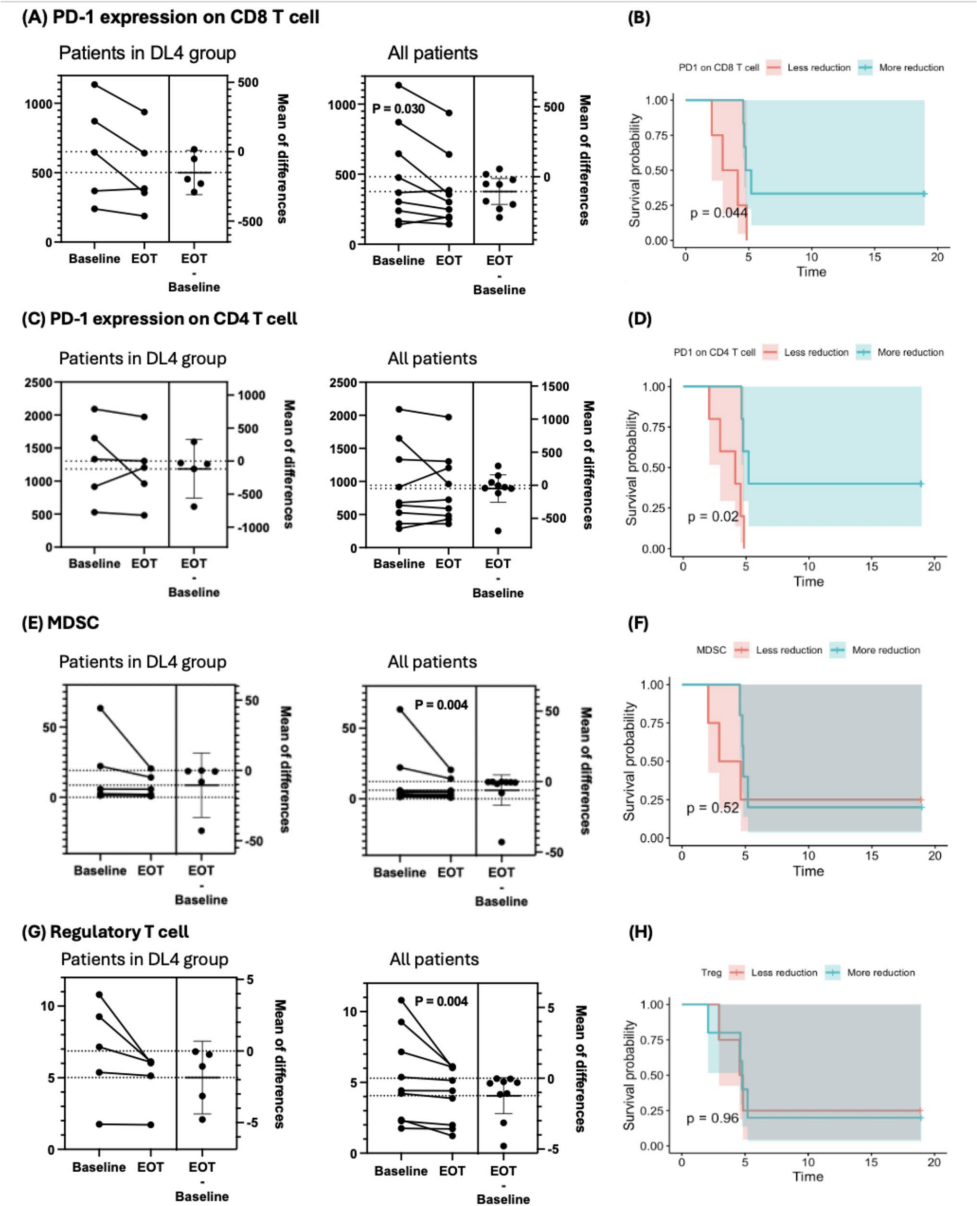
Comprehensive immunophenotyping using 21-color flow cytometry and corresponding survival analysis. Among the 21 trial participants, only 9 patients were able to provide paired pre-treatment (baseline) samples with post-treatment (EOT) samples, which were evaluated in this section. **A.** PBF-1129 treatment led to a significant reduction in PD-1 expression on CD8⁺ T cells, indicating a potential reversal of T-cell exhaustion. **B**. Patients with greater reductions in CD8⁺ PD-1 expression showed a modest improvement in OS (median OS: 4.98 months; 95% CI, 4.63-NR) compared to those with lesser reductions (median OS: 3.53 months; 95% CI, 2.07-NR; *P* = 0.044). **C**. While PD-1 expression on CD4⁺ T cells did not change significantly overall, **D**. patients with greater reductions demonstrated significantly prolonged OS (median OS, 5.20 months; 95% CI, 4.77-NR) compared to those with lesser reductions (median OS, 4.13 months; 95% CI, 2.93-NR; *P* = 0.020). **E,G**. Treatment with PBF-1129 significantly reduced the proportion of MDSCs and Tregs in peripheral blood, suggesting an immunostimulatory effect. However, these changes were not associated with differences in OS **F, H**

Assessment of PD-1 expression on CD8⁺ T cells revealed a significant reduction (*P* = 0.03) following PBF-1129 treatment, suggesting a potential alleviation of T-cell exhaustion (Fig. 3A). Patients exhibiting greater reductions in PD-1 expression on CD8⁺ T cells experienced a modest improvement in OS (median OS, 4.98 months; 95% CI, 4.63-NR) compared to those with lesser reductions (median OS, 3.53 months; 95% CI, 2.07-NR; *P* = 0.044) (Fig. 3B). Although no significant changes in PD-1 expression on CD4⁺ T cells were observed before and after PBF-1129 treatment (Fig. 3C), patients with greater reductions (count reduction of > 15/µl) demonstrated significantly prolonged OS (median OS, 5.20 months; 95% CI, 4.77-NR) compared to those with lesser reductions (median OS, 4.13 months; 95% CI, 2.93-NR; *P* = 0.020) (Fig. 3D).

In contrast, the proportion of MDSCs in peripheral blood significantly decreased (*P* = 0.004) following PBF-1129 treatment, suggesting an immunostimulatory effect (Fig. 3E); however, this reduction was not associated with differences in OS (Fig. 3F). Similarly, the proportion of regulatory T cells (Tregs) in peripheral blood also significantly decreased (*P* = 0.004) post-treatment but was not correlated with OS (Fig. 3G and H).

Furthermore, reductions in PD-1 expression on CD8 + T cells and the percentage reduction of MDSCs demonstrated a trend toward improved tumor response. Additionally, post-treatment analysis revealed a trend of reduction in the expression levels of the adenosine-generating CD73 ectoenzyme on both macrophages and MDSCs (Supplementary Figure S3A and S3C). However, the extent of this reduction was not associated with OS (Supplementary Figure S3B and S3D) or tumor response. One patient in the highest dosage group entered the trial with the highest pre-treatment proportion of MDSCs at 62.4%, which decreased by approximately two-thirds to 19.7% after 1 treatment cycle. Although this patient achieved only SD as the best response, the patient had an OS of over 1.5 years from trial entry.

## Discussion

We report the results of a dose-escalation trial evaluating PBF-1129, an oral small-molecule A2BAR inhibitor in 21 patients with heavily pretreated, ICI-resistant metastatic NSCLC. PBF-1129 was well tolerated up to the maximum planned dose of 320 mg daily. The most commonly observed toxicities were gastrointestinal (nausea, vomiting, anorexia, diarrhea) and fatigue. Grade ≥ 3 TRAEs were uncommon which suggests a safety profile compatible with chronic administration and future combination strategies. Furthermore, we identified changes in peripheral blood immune cells including a reduction in PD-1 expression on CD8 + and CD4 + T cells and a decrease in the percentage of peripheral MDSCs.

Despite advancements in immunotherapy, the response rate to ICIs remains low, and acquired resistance develops in most patients [25]. Immune cell dysfunction within the TME, involving various immune cells such as CD8 + T cells and natural killer (NK) cells, is widely recognized as a key factor contributing to immunotherapy resistance [26]. Beyond lymphocyte suppression by tumor-derived cytokines, preclinical studies have demonstrated that inhibiting adenosine receptors can enhance the antitumor activity of CD8 + T cells and NK cells [12, 27].

Emerging evidence has highlighted the role of adenosine and its receptor pathways in regulating the abundance of MDSCs within the TME. Both MDSCs and Tregs contribute to the generation of extracellular adenosine [19, 28], which promotes further recruitment and expansion of MDSCs through activation of A2BAR [20, 29]. This recruitment of MDSC has also been implicated in the upregulation of PD-1 expression on CD8⁺ T cells, a marker of T-cell exhaustion [30]. Clinical results with adenosine-pathway agents in NSCLC have been mixed to date. A2A receptor antagonists, AZD4635, have shown acceptable safety but limited activity as single-agent and combination therapy with PD-L1 inhibitor in lung-cancer cohorts [31]. In contrast, dual checkpoint combinations incorporating TIGIT plus PD-1 blockade with or without dual A2A/A2B inhibition, etrumadenant, improved outcomes vs PD-1 blockade alone in the randomized phase 2 ARC-7 trial of PD-L1-high, first-line metastatic NSCLC [32]. However, the triplet that added adenosine blockade performed similarly to the dual TIGIT and PD-1 blockade, leaving the additional clinical benefit of adenosine blockade in this setting uncertain [32]. In the CD73 axis, the phase 2 COAST platform trial suggested that adding the anti-CD73 antibody oleclumab to durvalumab as consolidation after chemoradiation increased ORR and prolonged PFS versus durvalumab alone in unresectable stage III NSCLC [33], a signal now being tested in phase 3 (PACIFIC-9). The mixed results highlighted the importance of biomarker-guided treatment decision. In the present study, PBF-1129 is consistent with the favorable tolerability reported for A2B inhibition and the relevant biomarker analysis supports further evaluation in rational combinations (e.g., with immune checkpoint inhibitors) where adenosine-rich, myeloid-inflamed tumors may be most susceptible. In this study, treatment with PBF-1129 resulted in a significant reduction in both MDSCs and Tregs. Notably, a marked decrease in PD-1 expression on CD8⁺ T cells was also observed following treatment, suggesting a potential reversal of T-cell exhaustion. Although no objective clinical responses were documented, several patients experienced prolonged survival. PK studies showed that the RP2D of 320 mg daily achieved levels that remained above the IC_90_ for the A2B receptor for 24 h. The favorable safety profile of PBF-1129 observed in this trial supports its further investigation in combination strategies aimed at enhancing antitumor immunity.

## Limitations

Limitations of this phase 1 trial include its single-site, non-blind, non-randomized nature, as well as heterogeneity of the patient cohort. A limitation of the correlative studies is the absence of tumor tissue profiling of T cells and MDSCs within the TME. Such profiling would help clarify whether the observed immune effects in peripheral blood are related to immune cell trafficking into the TME or direct alterations in peripheral blood immune cell phenotypes.

PBF-1129 was safe and tolerable in patients with heavily pretreated mNSCLC, although no responses were observed. The RP2D was identified as 320 mg orally daily. Given the potential of PBF-1129 to modulate antitumor immunity, these findings support the ongoing clinical trial investigating the combination of nivolumab (an ICI) and PBF-1129 (ClinicalTrials.gov: NCT05234307).

## Supplementary information

Below is the link to the electronic supplementary material.

ESM 1 (DOCX 1.28 MB)

## Data Availability

Data is provided within the manuscript or supplementary information files.
