# Supplementary material for: Phase 1 dose escalation trial of the selective adenosine A2B antagonist PBF-1129 in patients with metastatic non-small cell lung cancer
Source: Invest New Drugs. 2025 Dec 3;43(6):1211–21. doi: 10.1007/s10637-025-01591-y (PMC12753538; doi:10.1007/s10637-025-01591-y)
Supplement: Supplementary file 1 — (DOCX 1.28 MB) [file 10637_2025_1591_MOESM1_ESM.docx]

**Supplementary Files**

**Table S1. Flow cytometry panel**

| Lymphoid panel | | Myeloid panel | |
| --- | --- | --- | --- |
| Color | **Marker** | **Color** | **Marker** |
| Zombie NIR | Viability | Zombie NIR | Viability |
| BV421 | CD279 (PD-1) | BV510 | CD33 |
| SV500 | CD8 | BV650 | CD11c |
| BV605 | CD197 (CCR7) | BV711 | CD39 |
| FITC | CD44 | BV785 | CD73 |
| PE/Dazzle594 | CD62L | SB550 | CD11b |
| PE/FIRE640 | CD45RA | PE | CD203c (E-NPP3) |
| PE/FIRE700 | CD127 (IL-7Ra) | PerCP | CD16 |
| APC | CD25 | PE/CY7 | CD209 (DC-SIGN) |
| ALEXA F700 | CD4 | PE/FIRE810 | CD86 |
| APC/FIRE750 | CD3 | APC/FIRE810 | HLA-DR – APC function |

**Figure S1. Additional Kaplan-Meier survival analysis based on patient demographics. A.** No significant differences in OS were observed based on age difference (≥ 60 vs. < 60 years). **B.** No significant differences in OS were observed based on gender difference. **C.** No significant differences in OS were observed based on different PD-L1 expression levels (≥ 50% vs. < 50%). **D.** Patients with adenocarcinoma histology have a significantly longer OS than patients with non-adenocarcinoma histology.

**
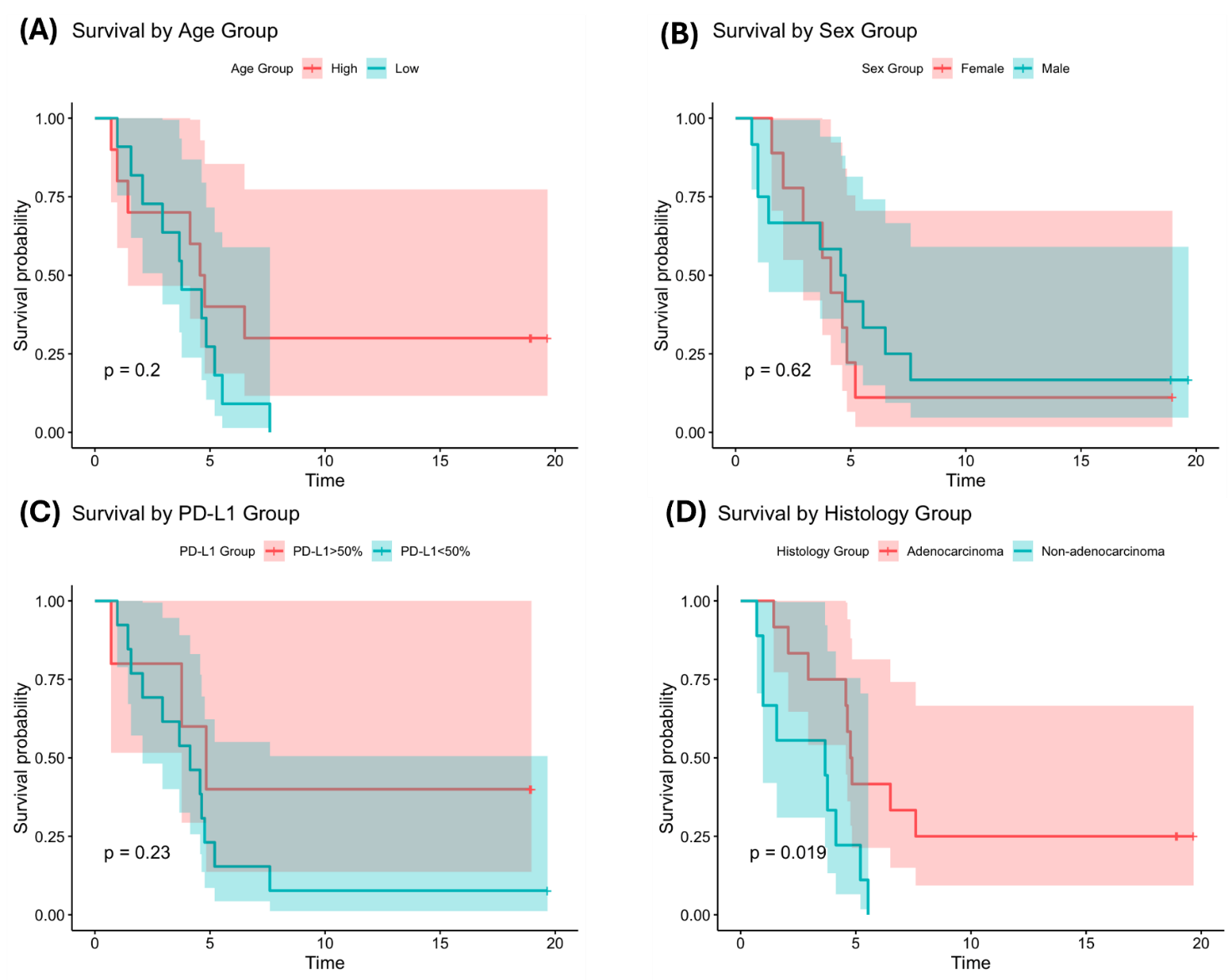
**

**Figure S2. Survival analysis based on patient plasma cytokine baseline. A.** Patients with higher IL-4 baseline (> 2.12 pg/ml) had a significant OS advantage than patients with lower IL-4 baseline. **B.** Patients with higher TNF-α (> 0.76 pg/ml) had a significant OS advantage than patients with lower TNF-α baseline. **C-G.** Patient baseline expression levels of IP-10, MCP-1, IL-17A, IL-6, and IL-2 were not associated with survival benefit.

**
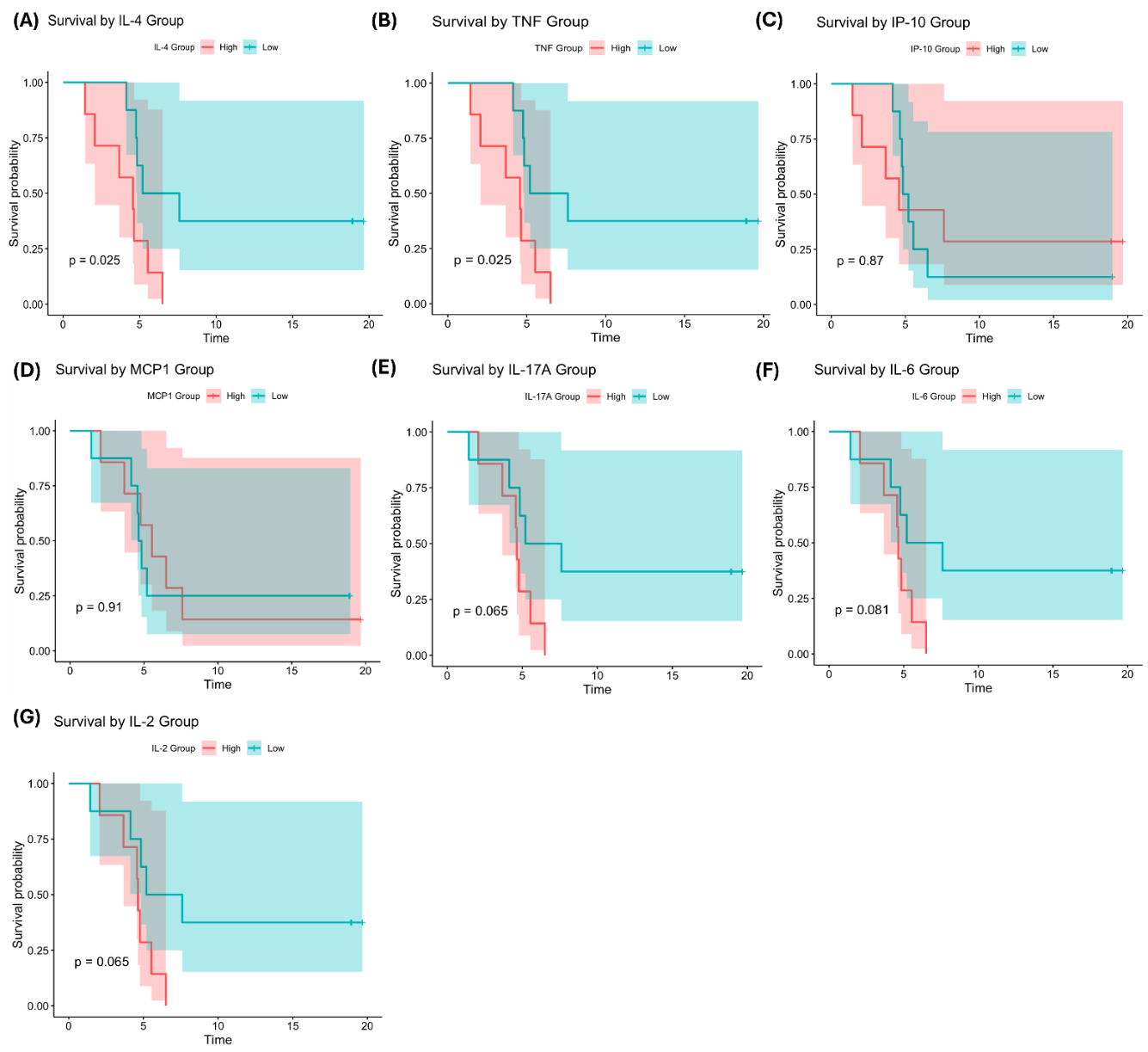
**

**Figure S3. Adenosine-generating ectoenzyme CD73 expression level evaluated with flow cytometry and corresponding survival analysis.** Among the 21 trial participants, only 9 were able to provide paired pre-treatment (baseline) and post-treatment (EOT) samples, which were evaluated in this section. **A.** PBF-1129 treatment led to a reduction of CD73 levels on macrophages, but this change was not associated with survival benefit (**B**). **C.** PBF-1129 treatment led to reduction of CD73 levels on MDSC, but this change was not associated with survival benefit (**D**).

**
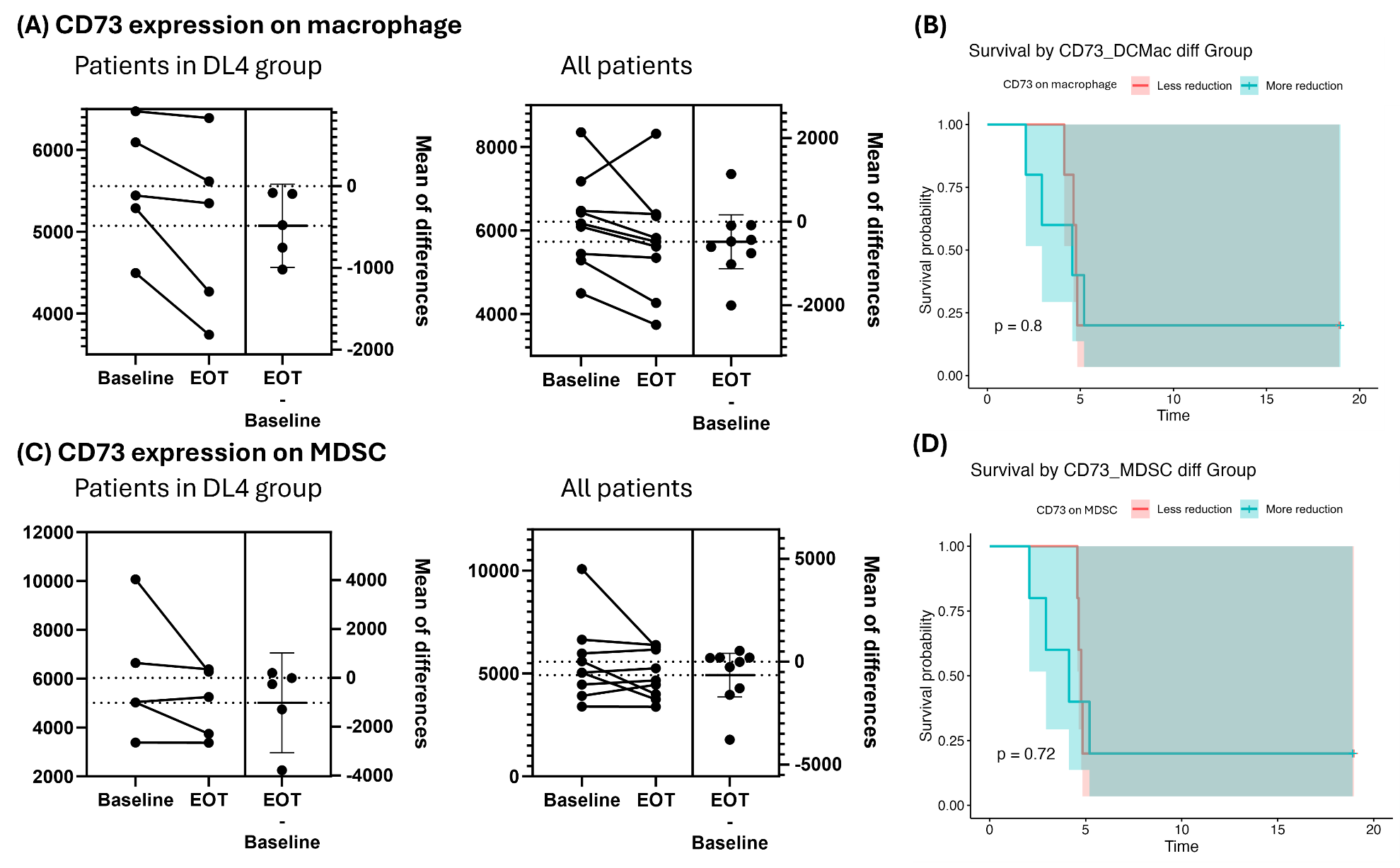
**
